# Recommendations from the Council of Emergency Medicine Residency Directors: Osteopathic Applicants

**DOI:** 10.5811/westjem.2018.9.39814

**Published:** 2018-11-19

**Authors:** Megan Stobart-Gallagher, Liza Smith, Jonathan Giordano, Zach Jarou, Lucienne Lutfy-Clayton, Adam Kellogg, Emily Hillman

**Affiliations:** * Einstein Medical Center Philadelphia, Einstein Healthcare Network, Department of Emergency Medicine, Philadelphia, Pennsylvania; † Baystate Medical Center, University of Massachusetts Medical School-Baystate Health, Department of Emergency Medicine, Springfield, Massachusetts; ‡ University of Texas Health Science Center Houston, McGovern Medical School, Department of Emergency Medicine, Houston, Texas; § University of Chicago, Department of Medicine, Biological Sciences Division, Section of Emergency Medicine, Chicago, Illinois; # Truman Medical Center, University of Missouri-Kansas City School of Medicine, Department of Emergency Medicine, Kansas City, Missouri

## Abstract

The Council of Emergency Medicine Residency Directors (CORD) Advising Students Committee (ASC-EM) has previously published student advising recommendations for general emergency medicine (EM) applicants in an effort to disseminate standardized information to students and potential advisors. As the shift to a single graduate medical education system occurs by 2020, osteopathic students will continue to represent a larger portion of matched EM applicants, but data shows that their match rate lags that of their allopathic peers, with many citing a lack of access to knowledge EM advisors as a major barrier. Based on available data and experiential information, a sub-group of ASC-EM committee sought to provide quality, evidence-based advising resources for students, their advisors, and medical leadership. The recommendations advise osteopathic students to seek early mentorship and get involved in EM-specific organizations. Students should take Step 1 of the United States Medical Licensing Exam and complete two EM rotations at academic institutions to secure two Standardized Letters of Evaluation and consider regional and program-specific data on percentage of active osteopathic residents.

## BACKGROUND

Historically, there have been two paths for the osteopathic student pursuing emergency medicine: osteopathic-affiliated residency programs through the American Osteopathic Association (AOA) match or Accreditation Council for Graduate Medical Education (ACGME) residency programs through the National Resident Matching Program® (NRMP®). In February 2014, the ACGME, AOA and American Association of Colleges of Osteopathic Medicine announced a path toward formation of a single graduate medical education accreditation system. Under this plan, AOA-approved programs must apply for ACGME accreditation by June 2020, at which time the AOA will cease all primary accreditation activities.^3^ As of May 2018, 50 of the prior 62 AOA programs have completed the ACGME accreditation process, bringing the total number of ACGME-accredited emergency medicine (EM) programs to 231. This process did lead to the closure of a handful of AOA programs that did not seek ACGME accreditation, decreasing the overall number of residency spots available to osteopathic candidates in traditional AOA programs.^3^ A 2017 survey of program directors from the American College of Osteopathic Emergency Physicians showed that a handful of their programs are also planning to pursue “osteopathic recognition,” reflecting their intention to maintain an osteopathic-focused learning environment.^4^, ^5^

**Figure 1:**
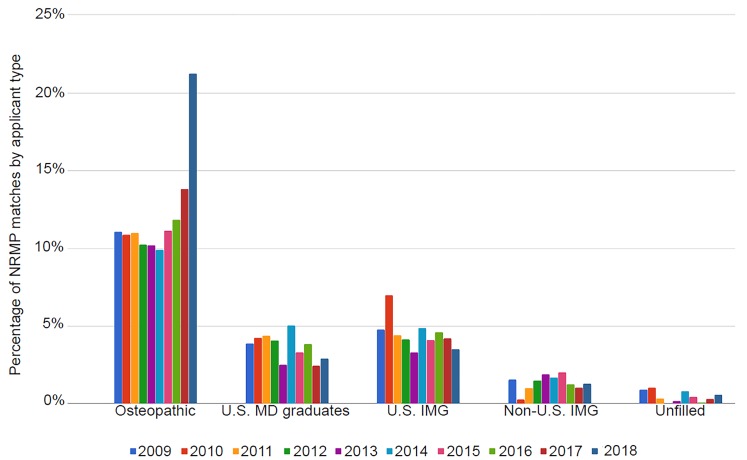
Longitudinal data from the National Resident Matching Program® showing the percentage of match positions secured by types of applicants who are not allopathic U.S. seniors. (U.S. MD graduates are those re-entering the match).^8^ *IMG*, international medical graduate.

The popularity of EM as a specialty has been growing over the last few years, and with the single graduate medical eduation (GME) accreditation system under the ACGME we are seeing a greater number of osteopathic students entering the overall pool of applicants. This will increase the overall number of applicants to each program and increase the competitiveness for osteopathic students. Over the last two years of match data, available spots for osteopathic students specifically have decreased. In 2017 there were 51 AOA programs that participated in the National Matching Services Inc. (NMS) match with 310 available; but in the 2018 match only 30 programs participated with 172 spots available specifically for osteopathic students.^6^ These students are now entering the NRMP match with competitive allopathic counterparts. In 2018, 81% of osteopathic applicants achieved a successful match in EM compared to 91% of their allopathic counterparts.^7^ Figure 1 shows the percentage of osteopathic as well as non-traditional United States (U.S.) allopathic graduates matched over time.^2^ Between the years 2009 and 2016, a stable 10–12% of osteopathic seniors matched into ACGME EM programs with a jump noted in 2017 and 2018 due to the aforementioned transition of AOA- accredited programs to the ACGME.^7^,^8^

Less than one-third of osteopathic medical schools (11/39) note affiliations with EM residency training programs on their respective websites; as a result, osteopathic students may not have ready access to EM program leaders for guidance. A recent study showed that 70% of current EM residents from allopathic schools had EM-specific mentorship available, but only 20% of osteopathic graduates had EM-specific mentorship available.^2^ These students must take the initiative to seek out those familiar with the ACGME match process, even if it means looking outside their home institutions. Many prospective applicants find an advisor late or do not have an advisor before the application process.^9^

## OBJECTIVES

The goals of the CORD ASC-EM in creating these recommendations are to provide consensus, evidence-based advice specifically for osteopathic students pursuing an ACGME-accredited residency and to equip advising faculty with the knowledge and resources to provide high-quality guidance.

## CURRICULAR DESIGN

To develop best-practice advising information for osteopathic students, an ASC-EM Osteopathic Advising Team was formed from members self-selected based on interest and expertise. The team was made up of 12 faculty members with specific interest in advising osteopathic students as well as residents. The faculty and residents ranged in experience from two years to over 20 years participating in both ACGME and AOA residency leadership. The team included program directors, EM residents, clerkship directors, core faculty members, medical education fellows, and chief residents from across the country. Much of the group hailed from the Northeastern region of the U.S., but there was also representation from Colorado, Michigan, Missouri, and Texas. One-third of the group are practicing osteopathic physicians. Additionally, osteopathic students worked with faculty to provide their input.

Development of the consensus recommendations was by collation of available literature, existing advising resources, active ongoing research and experience. Literature included available NRMP data and the AOA’s guide to the single GME accreditation system.^10^ It also included non-published documents created by individual medical student interest groups and residencies, many of which were creating their own recommendations based on the NRMP data and experience. Most of the formal advising resources were from the Emergency Medicine Residents’ Association (EMRA), Society for Academic Emergency Medicine, and the Association of American Medical Colleges websites. These sources were catalogued and researched for the evidence behind them vs. those noted as experiential in nature. Those with clear evidence were more highly regarded, while the experiential sources were thought to be areas for further research.

Drafted recommendations by the ASC-EM Osteopathic sub-group were made available for commentary to the entire ASC-EM, a group of 40 members representing EM faculty and residents across the country. Members were given several weeks to provide feedback on a shared Google document. The recommendations were also available for commentary to the entire CORD community and the public on the CORD blog.^11^ Feedback was received from members as well as a senior vice president for education within the AOA. We were contacted directly by administrators from the National Board of Osteopathic Examiners (NBOME). Subsequent communications between AOA and NBOME leadership with our team fostered a multi-faceted and collaborative approach for advising osteopathic students. Our recommendations remain on the CORD website and blog for continued commentary.

The following recommendations are offered as best-practice advising for osteopathic students and are intended to serve as a general guide, as each student needs an individualized approach.

**Figure 2:**
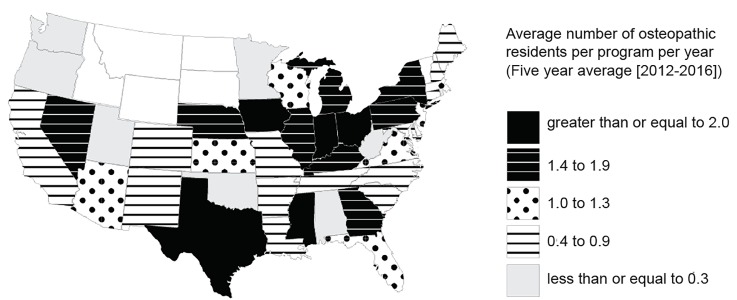
The geographic distribution of NRMP-matched osteopathic medical school graduates per ACGME-accredited emergency medicine residency program between the years 2012 and 2016.^22^ *NRMP*, National Resident Matching Program; *ACGME*, Accreditation Council for Graduate Medical Education.

1. **Pre-Clinical Years:** Students who have identified an interest in EM should seek out advisors within academic EM even if that means outside their own institution. If able, they should be active in their school’s Emergency Medicine Interest Group (EMIG) or chapter of the American College of Osteopathic Emergency Physicians resident-student section (ACOEP-RSO). For students at institutions without an EMIG or EM faculty advisors, we recommend that they consider joining the EMRA Student Council, where students can request to be paired with volunteer mentors anywhere in the country.^11^ They can also participate in monthly, virtual advising sessions using EMRA Hangouts where ASC-EM members regularly provide advice.^13^ The ASC-EM has also put together an osteopathic student-specific planner for both pre-clinical and clinical years, which is easily accessible on its website.^14^
2. **Comprehensive Osteopathic Medical Licensing Examination (COMLEX) and U.S. Medical Licensing Examination (USMLE):** Osteopathic students are required to take the COMLEX levels 1, 2CS/PE, and 3 as part of their education. To allow direct comparison to their allopathic peers, it is recommended that an osteopathic applicant take the USMLE exams (Step 1 and/or Step 2 Clinical Knowledge [CK]). A study from 2014 showed that osteopathic students who took the USMLE were more likely to successfully match at an ACGME program, and that 39% of program directors felt that taking the USMLE was extremely important.^15^ The NBOME does offer a percentile calculator to help transition the three-digit COMLEX score to a percentile rank. However, there is no accurate conversion of a COMLEX score to a USMLE score, which makes it difficult to provide direct comparison to the student’s allopathic peers.^16^–^18^ This challenge for ACGME programs may lead to some being unwilling to accept only a COMLEX score; this applies for both residency and medical student rotation applications.^19^ Students can use EMRA Match for specific program requirements. It is a web-based search created as a collaboration between EMRA, CORD, Clerkship Directors in EM (CDEM), and the American College of Emergency Physicians. It is updated regularly and has specific data on programs that prefer or accept only USMLE.^20^
  While taking both USMLE steps is ideal, taking at least Step 1 is preferable to not taking either. It is recommended that osteopathic applicants study specifically for the USMLE exam because, while it is similar to the COMLEX, there are enough differences that studying for one exam may not be sufficient preparation for the other. Programs will be more likely to grant interviews to students with a USMLE Step 1 score >235 or Step 2 CK score > 240.^19^, ^20^ Any student (osteopathic and allopathic) with a Step 1 score < 220 may have difficulty obtaining interviews and should be encouraged to take Step 2 CK early, to allow for results to be included in their initial application or begin to consider back-up plans in consultation with a faculty advisor.^19^
3. **Emergency Medicine Rotations:** Students should aim to perform two EM rotations during the summer or early fall months of their senior year at institutions with ACGME training programs in order to obtain standardized letters of evaluation (SLOEs) prior to the opening of the Electronic ResidencyApplication Service (ERAS®). The student should complete a home rotation if the school hosts an academic EM program, along with two visiting away rotations if no home rotation is offered. Many academic programs use the Visiting Student Learning Opportunities website to schedule audition electives, and begin accepting applications in early March. Some programs will have program-specific application requirements, such as submission of a USMLE Step 1 score or a brief personal statement. While community EM months can be a great learning experiences and expose an applicant to how emergency physicians practice, these rotations will not assist an applicant to nearly the same degree as they are not able to generate a SLOE.
4. **Letters of Recommendation:** These are one of the most highly valued parts of the EM application to programs when selecting applicants to interview.^19^ The letters of recommendation that carry the most weight are in the SLOE format and come from residency program leadership.^21^ It is recommended that applicants obtain at least two SLOEs – one from each EM rotation, with at least one uploaded by the time ERAS opens. These letters carry substantially more weight than traditional letters because they provide context for direct comparison of the applicant to his or her EM-bound peers.
5. **Program Selection:** The biggest obstacle to an osteopathic student’s application to the ACGME system is perceived competitiveness. According to the 2018 NRMP Program Director Survey, 80% of traditionally allopathic programs will typically interview and rank osteopathic students, narrowing the number of programs available overall.^19^ An osteopathic applicant would be well served to look at the composition of an individual program’s current residency classes to see whether there are osteopathic students represented. Having only a few, or no, current osteopathic residents means that an applicant should be realistic about the lower likelihood of an interview. Another resource for finding programs hosting current osteopathic graduates is EMRA Match,^20^ which has a filter for sorting programs by the percentage of osteopathic students training there. Students might also benefit from focusing on geographical areas that have historically matched higher percentages of osteopathic medical school graduates. As shown in Figure 2, between the years 2012 and 2016, Indiana, Iowa, Mississippi, Ohio, and Texas matched the most osteopathic students per ACGME residency program per year.^22^
6. **Electronic Residency Application Service (ERAS®) Application:** Students should aim to submit their application as early as possible, after ERAS opens. Programs begin reviewing applications almost immediately and may begin offering interviews even before the Medical Student Performance Evaluations (previously known as the Dean’s Letter) are released on October 1.^19^ Based on the NRMP program director data, osteopathic applicants should apply to between 20–40 programs that have a track record of training osteopathic graduates. Applications to >40 programs is rarely warranted and leads to diminishing returns at increased personal cost.^23^ The “numbers question” of applications to be made is particularly individualized and is best discussed directly with an advisor familiar with the student and the EM application process.
7. **Rank List:** Applicants should apply with the goal of obtaining approximately 12 interviews in order to rank approximately 12 programs. Data show that overall applicants (allopathic, osteopathic, and independent graduates) who ranked nine programs had ~90% match rate in EM. Those with 12 or more programs pushed that match rate up to 95–99%.^22^, ^23^ Because programs rank the clear majority of applicants they interview and the considerations that go into creating the exact order of the match list are highly variable and institution specific, there are no meaningful guidelines specific to osteopathic applicants regarding the interview process itself. There is limited information as to what degree interviews may affect a student’s rank-list placement; thus, this is an area for further study when comparing programs that interview both osteopathic and allopathic graduates.

While no guidelines can exactly fit the needs of every student, these recommendations represent consensus, best-practice advice generalized to the majority of osteopathic applicants. Students are encouraged to seek out well-informed advisors to address his or her specific circumstances and application goals. These recommendations were developed based on the most recent available literature. Because these recommendations are largely informed by the experience and expertise of our committee members, they remain subject to inherent prejudice and bias. As with the original advising document and recommendations, the ASC-EM will be seeking approval of our advising resource from CORD, CDEM, and the American Academy of Emergency Medicine for this year’s updates. The ASC-EM also seeks to capitalize on these relationships to more widely disseminate and make available these application resources.

## IMPACT/EFFECTIVENESS

The CORD ASC-EM developed the above recommendations based on a perceived need for specific advising aimed at osteopathic applicants. As the applicant pool merges, both osteopathic and allopathic graduates will funnel toward the same ACGME-accredited programs. We hope that these consensus recommendations will educate the osteopathic students and their advisors to direct their energies appropriately to maximize their EM applications in preparation for the NRMP match. This resource, in addition to the original consensus guidelines for the general EM applicant, will hopefully contribute to a better-informed pool of applicants who will apply wisely, as well as to a decrease in the overall number of excess applications per student.
